# Social and structural vulnerability as a barrier in HIV and/or AIDS communication campaigns: Perceptions of undergraduate students at a South African tertiary institution

**DOI:** 10.4102/jamba.v10i1.407

**Published:** 2018-03-27

**Authors:** Olivia Kunguma, Andre Pelser, Perpetua Tanyi, Collins Muhame

**Affiliations:** 1 Disaster Management Training and Education Centre for Africa, University of the Free State, South Africa; 2 Department of Sociology, University of the Free State, South Africa

## Abstract

The multicultural nature of a higher academic institution comprising students from different backgrounds can either negatively or positively influence student behaviour. Students might engage in high-risk practices, which in turn can make them vulnerable to HIV infection. Higher academic institutions are then tasked with finding strategies that can help to reduce this risk and vulnerability to HIV and/or AIDS. However, there are many issues and barriers, both from the institution and students, which can impede the success of any communication strategy. The University of the Free State’s main campus was selected for this study. A sample of 402 students from a total of 17 591 undergraduate students participated in the study. A structured questionnaire was randomly distributed to the undergraduate students. The sample was compiled across all faculties, as well as on campus and off campus. A transact walk on campus with an observation checklist was also used for triangulation purposes. The observation checklist helped to collect data on the visibility of male and female condoms in toilet facilities, and HIV and/or AIDS information on noticeboards, bins, stationery, billboards, etc. The main finding indicated that students were not knowledgeable about HIV and/or AIDS campaigns rolled out on campus. To support this, the observational transact walk results indicated that there were no visible campaigns on campus. Also, problems with existing communication and organisational barriers were found not only with the students but also with the implementation office. This study recommends that the university needs to engage with the students by identifying the root cause of their vulnerability. The university should explore and make use of all the available resources for a successful intervention, thereby building students’ resilience in preventing HIV infection.

## Introduction

Several scholars have found that there is a general misconception regarding the risk of HIV and/or AIDS transmission (Nelson, Ojebuyi & Salawi 2016; Sisk et al. 1988; Vaghela 2015). This misconception relates to the fact that educated people such as students enrolled at a university are supposed to be more aware of the transmission risks, and therefore less likely to become infected by the virus. According to Limaye et al. (2013), there is common misinformation concerning HIV and/or AIDS, which hinders the effective awareness and acceptance of individual and societal behavioural change. However, the perception about educated people being more cognisant of HIV and/or AIDS is supported by a joint report by the United Nations Programme on HIV/AIDS (UNAIDS), the United Nations Population Fund (UNFPA) and the United Nations Development Fund for Women (UNIFEM) (2013), which state that educated women are more likely to know how to prevent HIV infection and to abstain from risky sexual activities (Nelson et al. 2016).

Offering an alternative view, United Nations Children’s Fund (UNICEF), UNAIDS and World Health Organization (WHO) (2002) argue that globally there is a lack of HIV knowledge, with high infection rates among youths aged between 15 and 24 years (Karim & Karim 2005:266). Hence, young people need to be targeted for HIV and/or AIDS education to decrease the transmission and stigmatisation of the disease. Young people in many parts of the world, and specifically those in the above mentioned age group, are at high risk of HIV and/or AIDS infection. This is a result of unprotected sex, sex among men, alcohol and drug abuse and engaging in risky behaviours (Maimaiti et al. 2010).

Many students engage in high-risk practices, which render them vulnerable to HIV (Mutinta & Govender 2012; Ntozi & Odwee 2003:103; Shiferaw et al. 2014). According to Mazibuko (2000:1), Aggleton, Ball and Mane (2006:1) and Merkinaite, Grund and Frimpong (2009:113), globally, youths use drugs and alcohol for relaxation and fun to deal with inhabitation; to cope with pressure and frustration; to relieve stress, anxiety or pain; and to overcome boredom. Students in South Africa are not exempt from these practices. There is also an urge among young people to experiment, where they would engage in sexual intercourse without protection or engage in oral sex (Centers for Disease Control and Prevention 2009; Hops et al. 2011:2). In most cases, after being intoxicated with either drugs or alcohol, students are more likely to forget or be reluctant to wear a condom, or to insist on the partner doing so.

A vulnerability to and stigma of HIV and/or AIDS are inseparable factors that can impede HIV and/or AIDS campaigns (Mahajan et al. 2008:1). A fear of stigmatisation often prevents people from seeking treatment for HIV and/or AIDS or from admitting their HIV and/or AIDS status publicly. Stigma causes denial, which results in people neglecting to protect themselves. Studies carried out by Bamidele et al. (2012) among university students in India, South Africa and the United States on attitudes towards people living with HIV and/or AIDS (PLWHA) discovered that negative attitudes may reduce people’s willingness to get tested, thereby increasing the risk of HIV and/or AIDS transmission. Some scholars have proved that stigma causes people with HIV and/or AIDS to be mistakenly seen as some kind of problem rather than part of the solution (Association of Volunteer Emergency Response Teams [AVERT] 2016; Pop, White & Malow 2009).

According to Kushal (2011) and Keyton (2011), communication is the process of transmitting information from a sender to a receiver and, in most cases, miscommunication is encountered. This miscommunication or barrier occurs when what was intended to be communicated does not get communicated and subsequently a distorted message is transmitted. Kushal (2011) further identifies six sources of miscommunication: problems with the message formulation, difficulty in expressing ideas, transmitting the message, receiving the message, interpreting the message and differences between the sender and the receiver such as age and race. Several types of communication barriers are identified by Kushal (2011), Lunenburg (2010) and Parker et al. (2002), which include the following barriers: semantic or language, organisational, personal, emotional or perceptional, physical, societal, community, social network and individual barriers. Social network barriers, which involve partner or friendship support, as well as marketing and interpersonal communication. All the these were of interest in this study.

HIV and/or AIDS campaigns depend highly on the palatability of the advocated behaviour, the receptivity of the target audience and the quality and quantity of the messages (French et al. 2014). A possible barrier to communicating about HIV and/or AIDS is the stigma associated with the disease; for example, a student can deny applicability to self, with reference to recommendations like abstinence which can be unappealing and unacceptable to some students who assume the position of freedom once enrolled at university (Atkin, Rice & Ronald 2012). It is further noted that students at universities experience increased freedom from parental and school controls. For this particular study, it was argued that communication barriers to HIV and/or AIDS campaigns could materialise from students’ attitudes towards the virus and any messages about it.

Mberia and Mukulu (2011) believe that there is a need to discover more persuasive factors that influence the response of youths to HIV and/or AIDS campaigns. In agreement with this, Parker et al. (2002) support the need for an integrated communication strategy, which takes into consideration the range of media options available. These include approaches that involve small media, dialogue and participation. It is further argued that these approaches contribute more favourably to a behavioural change through supporting activities at the grass-roots level (Parker et al. 2002). Moreover, stigmatisation, a lack of social support and vulnerability are aspects often neglected in HIV and/or AIDS research and need to be discussed more.

The chosen study area, the University of the Free State (UFS), has three campuses all located in the Free State province of South Africa. The Main and South campuses are both in the city of Bloemfontein in the Mangaung Metropolitan Municipality, while the QwaQwa campus is located in the Thabo Mofutsanyane District Municipality. The UFS is a multicultural institution that hosts approximately 33 000 students on its three campuses. At the time of this study, about 28 186 students were studying at the main campus, 874 at the South campus and 4260 at the QwaQwa campus (Department of Communication and Brand Management 2017). This study was carried out at the main campus in Bloemfontein with the primary aim of determining students’ knowledge and perceptions about HIV and/or AIDS campaigns on campus. The knowledge and perceptions would then help to determine any possible communication barriers to the successful implementation of campaigns.

### Problem statement

One of the four key strategic objectives of the South African National Strategic Plan (NSP) for HIV and/or AIDS, sexually transmitted infections (STIs) and tuberculosis (TB) was to address social and structural barriers that increased the vulnerability to HIV, STI and TB prevention, care and impacts (ROSA 2012–2016). Communication was identified as the key strategic enabler that underpinned the entire NSP and which needed to determine the successes of its implementation. The communication enabler needed to include governance and institutional arrangements, effective communication, monitoring, evaluation and research. Thus, effective communication was viewed as critical for the implementation of the NSP, while communications regarding social and behaviourial change were also viewed as critical to changing risky behaviours and social conditions which drove the HIV and TB epidemics. As a result, the South African Government expanded its menu of options across the continuum of care from prevention, treatment, care and support to addressing the social drivers of ill health as well as locating the NSP into the broader development of the government’s agenda (ROSA 2012–2016).

Narrowing the policy discussion to higher academic institutions, studies conducted between 2008 and 2009 show that many higher institutions of learning in South Africa are affected by HIV and/or AIDS (HEAIDS 2010). As a result, responses at higher education institutions to the HIV and/or AIDS epidemic were mostly in the form of the implementation of policies and programmes. However, the communication barriers to these programmes are unknown and this has constrained planning processes.

Most higher education institutions within the Southern African Development Community (SADC) occupied a unique position to shape, debate and create actions and policies on HIV and/or AIDS. These institutions realise that in the absence of a cure, education is the best social response to the epidemic. Notably, other institutions of higher learning are not exempt from the fight against the spread of HIV and/or AIDS infection. Universities in Kenya, such as the Daystar University, host Voluntary Counselling and Testing (VCT) services for both students and employees. They believe that strategies like these are a milestone towards stigma reduction (Mwangi et al. 2014). Other universities like the University of Zululand integrated HIV and/or AIDS information and education in their orientation programme for first-year students. It is also mandatory for each faculty to design a specific HIV and/or AIDS module, which would fit in to that faculty’s specific programmes. The inclusion of such a module serves to equip students to manage the epidemic while still at university and after they have graduated. This process was evaluated and monitored through reports from the heads of departments to the respective deans and then incorporated into annual faculty reports. Subsequently, the universities’ ongoing HIV and/or AIDS awareness strategy mandated that they embark on awareness campaigns on specific occasions such as Valentine’s Day and World AIDS Day. Other activities involved advice on treatment, which includes alternative therapies for opportunistic infections (Unizul 2010). The University of Zululand’s prevention communication strategy was to ensure that those individuals who would have tested HIV negative after the introduction of the VCT campaign would maintain their status.

The UFS has made an effort to communicate to its students about the virus and to come up with various solutions to keep students aware and protect them from the HIV and/or AIDS crisis. Already in 1990, the UFS established a conveniently located clinic and counselling centre on the main campus. The HIV and/or AIDS Wellness Centre has for the past 10 years led to the development of information campaigns and workshops targeting both students and staff members. These campaigns and workshops were introduced under many banners and initiatives: STI and Condom Week, the Voluntary Counselling and HIV Testing Campaign (VCHTC), the Jes Foord Breakfast, the Word-A-Thorn Crossword Competition, the Transformers Re-union at World AIDS Day, the Candlelight Memorial on World AIDS Day and the New Start Male Circumcision Campaign.

In 2001, a study described the HIV and/or AIDS plan of the UFS as socially responsible, because it supported condom distribution, counselling at a campus clinic and the consideration of education and changes to the curriculum (Van Wyk, Pieterse & Otaala 2006). However, an audit visit by Booysen, Bachmaan and Pelser (2005) established that although the VCHTC has been available to both students and staff since 1990, only 10–20 students and staff members make use of the initial free test per week. Although the need for such a type of service might be high, there is a possibility that the stigma or fear associated with HIV and/or AIDS prevents greater numbers of students and staff from coming forward. The audit panel was concerned that despite the activities of the UFS HIV and/or AIDS Unit, there was no visible campaign on HIV and/or AIDS awareness and prevention on the main campus. This ties in to another concern expressed by Mbengo (2013:1), namely, that despite the availability of VCT services in the majority of South African universities, most university students are still unaware of their HIV and/or AIDS status. A lack of knowledge of their HIV status puts these students and others at risk. The stigma or fear associated with HIV and/or AIDS is a personal, emotional or perceptional barrier. The lack of HIV and/or AIDS communication campaign visibility on campus is an organisational or marketing barrier.

### Theoretical framework

This study employed the social support theory to determine undergraduate students’ understanding of and attitude towards HIV and/or AIDS campaigns. Albrecht and Adelman (1987:1) define social support as either verbal or non-verbal communication between recipients and providers, helping to reduce the individual’s uncertainty about a situation. Other authors like Hunter (2011) define social support as a network of family, friends, neighbours and community members who are available in times of need to give psychological, physical and financial help. Hunter’s (2011) definition, compared to Albrecht and Adelman’s (1987) definition, accentuates the network of typical people who are available to provide support as well as the type of assistance that can be provided by a certain network. Scholars like Schaefer, Coyne and Lazarus (1981) continue to identify five types of social support, namely, emotional, esteem, network, information and tangible support.

This study also explored the different types of stigma that students could experience. According to Churcher (2013), there are four types of stigma. These are vicarious, perceived, enacted and internalised stigma. Vicarious stigma is explained as a disease where people fear infection and feel the virus is an illness of immorality. Perceived stigma is a result of what the community perceives HIV and/or AIDS to be. Enacted stigma is the stereotype related to the virus, such as HIV being high among drug users. Lastly, internalised stigma is a personalised endorsement of stigmatisation beliefs, even as a form of self-judgement as a result of social, cultural and religious beliefs. A well-researched and developed HIV and/or AIDS risk reduction measure takes all of the above into consideration.

Developing HIV and/or AIDS communication solutions requires some understanding of the theoretical models which should form the foundation of any campaign. Social support is an intercultural communication strategy which can be explored or used to strengthen an HIV and/or AIDS campaign. Social support was thus used in this study to explore the respondents’ coping strategies and needs.

### Research objectives

The research objectives were to investigate students’ knowledge about HIV and/or AIDS campaigns implemented at the UFS; to investigate the visibility and prominence of these HIV and/or AIDS campaigns on campus; to identify communication barriers and to find a solution to the communication barriers; to suggest mechanisms to address the existing campaigns; and to identify social and structural factors that render students vulnerable and erode the impact of HIV communication campaigns.

## Methodology

This study contained elements of both qualitative and quantitative research design approaches. A case study of undergraduate students at the UFS was used. From a quantitative angle, the study was designed around a Knowledge (K), Attitude (A) and Practices (P) survey by distributing a questionnaire. The qualitative component entailed a transact walk and, more specifically, observations using a checklist. The study was conveniently carried out on the UFS main campus since it houses the office of Health and Wellness. A multistage sampling approach was adopted for sampling purposes. The target population consisted of undergraduate students enrolled at the UFS and who, at the time of the study, totalled 17 591. This group consisted of students aged between 18 and 25 years. This age group was chosen because it is one of the most vulnerable age groups for contracting or being affected by HIV and/or AIDS. The final stage involved a non-random sampling (convenience) approach, which was based on the distribution of questionnaires at key points. The size of the sample was 402 and because of the non-probability design of the sample, the findings could not be extrapolated to the rest of the student population at the UFS.

For the transact walk, 21 residences were visited, of which 10 were female and 11 were male residences. A total of 24 Lecture halls, 30 historical buildings, 14 sports facilities, 15 recreational halls and some toilets in these facilities were visited. Other facilities or campus amenities such as notice-boards, bins, parking lots, billboards, UFS-branded vehicles, students’ clothing and stationery were also observed.

The questionnaire had five categories that were measured with a 5-point Likert scale, ‘yes’ or ‘no’ answers and open-ended questions. The five categories consisted of demographics and background questions, HIV and/or AIDS knowledge and awareness, HIV and/or AIDS stigma-related or attitude questions, HIV and/or AIDS behavioural practice, and social support questions. The students participated voluntarily and no personal questions about their HIV status were asked. The questionnaire was distributed over the course of a week and on different days at various busy points at the university. Places of distribution included the university’s entrance gates, taxi rank, student centre, faculty building entrances, on-campus residence entrances and lecturer hall entrances. Before handing out the questionnaire, the students were asked if they were undergraduates and upon confirmation a questionnaire was handed out to them. The observation checklist had five observation variables, which were visible HIV and/or AIDS billboards or information boards; HIV and/or AIDS-branded vehicles; the presence of HIV and/or AIDS flyers at building reception desks or any accessible information dispenser; the wearing of promotional materials related to HIV and/or AIDS such as T-shirts, caps and student backpacks; and the availability of male or female condoms in both male and female toilet facilities. The transact walk was carried out over 2 months and, if observed, the items were ticked off from the checklist in accordance with the area visited.

The data were analysed by means of descriptive statistics (means, frequencies, cross tabulations). The difference in means was used to analyse the descriptive characteristics. The observation checklists were captured in an Excel sheet according to the five observation themes. Some of the qualitative data from the observations were triangulated with the quantitative data from the questionnaire to ensure a higher accuracy of the data.

### Ethical considerations

The questionnaire was submitted for ethical clearance and also checked by the HIV and/or AIDS Unit for approval and confidentiality.

## Discussion of findings

From the total population that participated, 57% were females and 43% were males. A large group of respondents (79.19%) resided off campus; that is, either with parents, a partner, in a flat or in a student commune (J. Botha, pers. comm., 18 October 2017). The majority of the students were residing off campus; because it was more affordable than on-campus accommodation and free from the campus residences’ regulations and restrictions. There was a possibility that the degree of freedom that the undergraduate students enjoyed, whether they reside either on or off campus, might have contributed to their vulnerability to HIV infection.

### University faculties: HIV and/or AIDS modules in the curriculum

A study by Van Wyk et al. (2006), carried out at the UFS in 2001, highlighted that the UFS HIV and/or AIDS plan supported condom distribution, counselling at a campus clinic and, most of all, consideration of HIV and/or AIDS education changes to the curriculum. More than a decade after the release of this report, the institution has still not seen the effective implementation of this plan. The respondents were from various faculties at the university and it was found that less than 40% of the respondents from each faculty indicated that they had encountered HIV and/or AIDS teachings in their curriculum. This was an indication that the UFS HIV and/or AIDS plan had not been successfully implemented.

### Respondents’ perceptions about HIV and/or AIDS campaigns

The 402 respondents were asked if they were aware of nine campaigns which the HIV and/or AIDS Unit had claimed to have implemented throughout the year. Of these, 52 respondents indicated that they were aware of the STI and Condom Week, 47 were aware of the First Things First Campaign, 31 were aware of the Jes Foord Breakfast, 25 were aware of the Word-A-Thorn Crossword Competition, 46 were aware of the Transformers Re-union at World AIDS Day, 40 were aware of the Blue Light Campaign celebrated during World AIDS Day, 85 were aware of the Candlelight Memorial on World AIDS Day, 80 were aware of the New Start Male Circumcision Campaign and 149 were aware of the VCHTC.

Respondents who experienced the campaigns in a positive light mentioned that the campaigns were helpful in reducing and preventing infection. Further benefits of the campaigns as mentioned by 13% of the respondents were that they were taught to live with the virus, and that voluntary counselling helped students know their status as well as encouraged them to use condoms through the STI and Condom Week campaign. Few respondents (5%) indicated that they were encouraged to use social support services, like church, and to manage their studies. However, there were some negative perceptions about the strategies as well. Two-thirds (66%) of the respondents mentioned that the HIV and Wellness Unit was not doing its job, because of a lack of campaign visibility. Students who witnessed these campaigns said that they were not informative enough. Some respondents (45%) felt uncomfortable attending public HIV and/or AIDS activities. The students preferred the activities to be more private and confidential, with some urging that professionals instead of students be used for counselling. Most respondents (78%) felt that the campaigns needed to be advertised along with frequent condom distribution. Most respondents (89%) suggested that the campaigns should cover the early symptoms of HIV infection in order for them to seek help in time.

### Respondents’ perceived knowledge of HIV infection and risk

These findings addressed the research objective relating to factors that increased students’ vulnerability as well as the possible communication barriers that hampered the successful implementation of HIV and/or AIDS campaigns. Table 1 illustrates the students’ perceived mode of HIV virus transmission as well as those individuals perceived to be most likely to be infected.

**TABLE 1:** Perceived mode of HIV infection and those perceived to be highly at risk.

Students’ perceptions of mode of transmission of HIV and/or AIDS	*N* = 402	
	Yes (%)	No (%)
HIV is high among same-sex partners	49	51
HIV is high among drug abusers	69	31
HIV is high among those with multiple sex partners	96	4
A person can get HIV from blood donation under sterile precautions	20	80
HIV can spread through casual contact with a person with AIDS	13	87

These results clearly indicated the existence of misconceptions among the undergraduate student population when it comes to HIV and/or AIDS. Even more disturbing was the fact that 4% of the respondents did not perceive multiple sex partners to be a high-risk behavioural practice and possible mode of HIV infection. Sexual intercourse with multiple partners has been identified by (UNAIDS & WHO 2015) as a high-risk behaviour associated with HIV infection. A possible misconception, which might have led to stigma, was that HIV could spread through casual contact with a person with AIDS. In Table 1, 13% of the respondents agreed with this misconception, which was too high a number and called for more attention in the HIV and/or AIDS communication campaigns since such a belief could lead to the stigmatisation of others.

### Vulnerability factors to HIV and/or AIDS

As mentioned above, if students are vulnerable, then they are easily exposed to HIV and/or AIDS infection. Among the factors that usually increase people’s vulnerability to HIV and/or AIDS are poverty and gender inequality (UNAIDS & WHO 2015). Vulnerability components identified in this study are given in Table 2.

**TABLE 2:** Students’ perceived vulnerability to HIV and/or AIDS.

What in your perception are the factors that lead students to engage in risky activities that might make them vulnerable to HIV and/or AIDS?	Percentage (*N* = 402)
Gender-based inequalities and violence (fear/threats from sexual partner)	7
Poverty (lack of money)	20
Harmful cultural practices (e.g. arranged marriage)	1
Lack of campaigns focusing on same-sex relationships and transactional sex	5
Lack of campaigns focusing on transactional sex	6
Alcohol and drug use by students	39
Reluctance to disclose HIV status owing to actual or perceived stigma	13

Around 39% of the respondents were of the opinion that alcohol and drugs contributed to an increased vulnerability to HIV and/or AIDS. This was followed by 20% of the respondents who associated a lack of money with increased vulnerability to HIV and/or AIDS. The main source of income for some respondents (29%) was bursaries; however, bursaries are not easily accessible. Approximately two-thirds (62%) of the students’ expenditure per month was between R100.00 and R1500.00, which was not enough to sustain a student’s complete needs. A lack of sufficient income can expose students to social vulnerabilities, such as forcing them into sexual relations that might make them vulnerable to HIV and/or AIDS infection (UNICEF 2008). Thus, this root cause could be a barrier to the implementation of any HIV and/or AIDS programme.

### Respondents’ knowledge of HIV and/or AIDS prevention strategies

The respondents were asked if they agreed or disagreed with whether it was possible to reduce the chance of contracting HIV by (1) using a condom, (2) having one sexual partner, (3) getting circumcised and (4) abstaining from sexual intercourse. In response, 95% of the respondents agreed that using a condom would reduce HIV infection. Most respondents (83%) agreed that having no more than one uninfected sexual partner at a time could serve as a prevention strategy. With regard to clinical male circumcision, 79% said HIV infection could indeed be reduced in this way. Lastly, as far as abstaining from sexual activities was concerned, 88% of respondents were of the opinion that abstinence could reduce the risk of HIV. These findings point to relatively high levels of ignorance and a lack of knowledge among the respondents. At least 80% of the 171 male participants agreed with male circumcision as compared to their female counterparts (77% of 231), which was an indication that male students were taking control of their health and reducing their risk of infection (Table 3). A lack of knowledge in preventing HIV infection is seen as a component of vulnerability, termed by Israel, Lauder and Simonetti (2008:5–6) as individual vulnerability.

**TABLE 3:** Students perceived HIV and/or AIDS prevention knowledge and/or strategies.

Is it possible to reduce the chance of getting the HIV virus by doing the following?	Yes (*n* = 171; Male)		Yes (*n* = 231; Female)	
	*n*	%	*n*	%
Always using a condom	161	94	222	96
Having one uninfected sexual partner	150	87	184	79
Clinical male circumcision	138	80	180	77
Abstinence	145	84	210	90

The majority of both male (94%) and female (96%) respondents agreed with the use of condoms at all times as a preventative strategy (Table 3). However, it seemed that both male and female respondents needed more convincing through campaigns with regard to having one sexual partner, clinical male circumcision and abstinence as HIV and/or AIDS prevention strategies. According to WHO (2010), masculinity is believed to be one of the factors that encourage men to have multiple sexual partners. Moreover, abstinence might be the last thought on their mind with all the freedom and opportunities students are exposed to at university.

### Communication barriers to HIV and/or AIDS campaigns, vulnerability, perceived stigma and social support needs

According to Churcher (2013:2), people’s beliefs can lead to either a stigmatisation of others or self-stigmatisation. From the study, 69% and 49% of the respondents held the view that HIV prevalence was high among drug abusers and same-sex partners, respectively. This is known as perceived and enacted stigma (Churcher 2013). Furthermore, 58% of the respondents indicated that they preferred going to off-campus clinics to test for HIV or any other sexually transmitted disease so as to avoid being identified as a person with AIDS, regardless of their status result. Approximately two-thirds (64%) said they would never disclose their HIV status, owing to a fear of stigmatisation on campus. Another high proportion (51%) was of the opinion that if they were to visit the HIV and/or AIDS Unit on campus, other students would assume they have AIDS. Another 38% acknowledged that they were reluctant to visit the unit because they believed the health care workers lacked confidentiality. Half (50%) of the respondents felt that if they were to be infected with HIV, they would feel judged and blamed by other students. Discouragingly, 64% of the students indicated that, should they be on HIV treatment, they would not feel comfortable taking their medication among friends on campus. Evidently, this is an indication of the existence of stigma and how it can negatively affect any possible HIV and/or AIDS communication campaigns on campus. In this regard, an important dimension of the study was to identify the possible effects of stigma through inquiring about respondents’ reactions if they were to discover that they have AIDS.

These results suggested the presence of vicarious stigma, meaning that people perceived HIV to be an illness of immorality (Saki et al. 2015:3). To support this, the respondents were asked what their reaction would be if they were to find out that they have AIDS. Few respondents (6%) indicated that they would feel personal embarrassment and fear being shamed by their family and the community. Alarmingly, 11% said they would commit suicide and 10% said they would feel sad and hopeless. These responses indicated feelings of all kinds of stigma – enacted, internalised, perceived and vicarious.

Respondents were asked to opine on various statements related to social support. About 42% indicated that they felt there was no one available with which to share fears and private worries. Just more than half (57%) indicated that, if they were to fall sick while on campus, they would easily find someone to help them with their studies. About 64% of the respondents indicated that there was someone they could turn to for advice regarding their health care or treatment. Lastly, a high proportion (76%) indicated that when they need suggestions on dealing with personal problems, there was someone available to assist.

Respondents were asked to indicate their social support needs. About 10% indicated that provision should be made for young PLWHA, in the event that they missed lectures or exams. About 23% indicated that they wanted a better understanding and protocol around the disclosure of their HIV and/or AIDS status on campus. Education regarding all forms of stigma and discrimination on campus was the biggest need among 31% of the students. About 19% indicated that the role of the HIV and/or AIDS Unit should be strengthened in university communications. Lastly, 17% of the students indicated that various networks aimed at supporting young PLWHA on campus need to be established and be very active.

**FIGURE 1:**
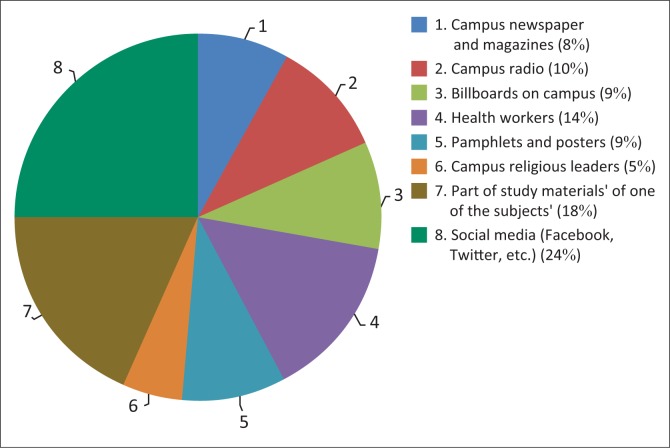
Preferred method of HIV and/or AIDS information communication.

The respondents also indicated their preferred method of communication. The UFS has a rich platform of communication channels which include a private radio station called ‘Kovsies FM’, a newspaper called ‘Irawa’, four billboards situated at the entrances to the university and which are also visible to the public, a Twitter account, a Facebook page and magazines. Figure 1 illustrates the respondents’ preferred methods of communication on related issues.

About 25% of the respondents preferred social media as a platform of communication on HIV and/or AIDS. Moreover, according to a research carried out on all UFS campuses by the Centre for Teaching and Learning (2013), almost all students use email, social networks, short message service (SMS) and Blackboard to communicate for academic purposes. Clearly, the HIV and/or AIDS Unit has vast resources which they can explore to effectively reach out to students. It is further significant that 19% of the respondents were in support of the inclusion of HIV and/or AIDS communication in their study materials.

### Observation results

The 2-week transect walks yielded no visible signs of any HIV and/or AIDS campaigns. The four billboards erected at the main entrances to the campus carried messages which welcomed everyone who stepped onto the campus. However, these billboards carried no messages about the university’s HIV and/or AIDS policy. HIV and/or AIDS risk reduction or prevention resources like condoms were non-existent at convenient places. There was the possibility that there were no campaigns running at the time of the transact walk, but at least past campaigns should have been visible with old posters on noticeboards and condoms should have been available at toilet facilities throughout the year. This lack of visibility of HIV and/or AIDS communication campaigns on campus indicated organisational and marketing barriers.

### Recommendations

The university needs to formulate a communication strategy in collaboration with the Student Representative Council that ‘speaks the language’ of students to make use of students’ social media platforms so as to repackage the message in order to appeal to the culture and social dynamics.

Through regular focus group discussions with the students, the university needs to find the most efficient channels that can avoid a top-down approach.

A university is an institution with many resources that can be explored and used to communicate a message of HIV and/or AIDS prevention and mitigation. As such, an academic institution is a good foundation for building resilience to HIV and/or AIDS infection through education and awareness. The following recommendations pertaining to the study on the UFS campus serve to illustrate this point.

The Health and Wellness Unit should extend their programmes beyond the campus and work hand in hand with off-campus residence owners, since 79.19% of the respondents in this study lived off campus.

HIV and/or AIDS education should be compulsory for all students in their first year of study, either as a UFS 101 subject or as part of one of the compulsory subjects. This should be encouraged by all the faculty deans and incorporated into the agenda of faculty board meetings.

Social media has become very popular not only among students but also among various organisations. Social media is an affordable and abundant resource; hence, the higher academic institutions should make use of it to educate students about HIV and/or AIDS prevention and coping mechanisms.

For any successful HIV and/or AIDS communication strategy, the root cause of HIV infection, barriers to the successful implementation of HIV and/or AIDS programmes and students’ social support needs must be identified.

The HIV and/or AIDS Wellness Unit on campus should partner with non-governmental organisations and governmental organisations working with HIV and/or AIDS for contributions and participation in mitigating and preventing HIV and/or AIDS among students.

## Conclusion

The study findings indicate that there are some strategic gaps in the HIV and/or AIDS prevention and mitigation interventions at the UFS. Various contributing factors serve as barriers to the success of HIV and/or AIDS campaigns at the university. These barriers manifested from the students’ perceptions about the risks of the virus, knowledge about the virus and strategies to protect themselves. The barriers also materialised from the UFS HIV and/or AIDS Unit’s weak risk reduction strategies. Financial investment into the survival of the unit needs to be visible to the recipients. In addition, it is not only about the development of an HIV and/or AIDS campaign but also about backing it up with needed resources and implementing it with an effective and integrated communication package. Possible weaknesses with HIV and/or AIDS campaigns, such as a lack of targeting, timing, monitoring and evaluation, fatiguing messages and the possibility of boring and uncreative campaigns, need to be identified. Furthermore, disregarding detailed vulnerability assessments for possible socio-economic problems that might lead to exposure to HIV and/or AIDS can affect the development and implementation of HIV and/or AIDS campaigns. Issues that affect the success of HIV and/or AIDS campaigns such as stigma, a weakness in the programme, a lack of social support, distorted perceptions and poor knowledge should be identified and addressed.
